# Reorganisation of primary care services during COVID-19 in the Western Cape, South Africa: Perspectives of primary care nurses

**DOI:** 10.4102/safp.v63i1.5358

**Published:** 2021-11-24

**Authors:** Talitha Crowley, Danine Kitshoff, Frances de Lange-Cloete, Justine Baron, Santel de Lange, Cornelle Young, Tonya Esterhuizen, Ian Couper

**Affiliations:** 1 Department of Nursing and Midwifery, Faculty of Medicine and Health Sciences, Stellenbosch University, Cape Town, South Africa; 2 Division of Epidemiology and Biostatistics, Department of Global Health, Faculty of Medicine and Health Sciences, Stellenbosch University, Cape Town, South Africa; 3 Ukwanda Centre for Rural Health, Department of Global Health, Faculty of Medicine and Health Sciences, Stellenbosch University, Cape Town, South Africa

**Keywords:** COVID-19, nurses, primary health care, services reorganisation, consequences of reorganisation

## Abstract

**Background:**

Primary care nurses play a pivotal role in the response to disasters and pandemics. The coronavirus diseases 2019 (COVID-19) pandemic required preventative, diagnostic, and curative measures for persons presenting with symptoms of COVID-19 by healthcare providers, whilst continuing other essential services. We aimed to investigate the reorganisation of primary care services during COVID-19 from the perspectives of primary care nurses in the Western Cape province of South Africa.

**Methods:**

We administered an online survey with closed and open-ended questions to professional nurses enrolled for a Postgraduate Diploma in Primary Care Nursing at Stellenbosch University (2020) and alumni (2017–2019) working in the Western Cape. Eighty-three participants completed the questionnaire.

**Results:**

The majority of the participants (74.4%) reported that they were reorganising services using a multitude of initiatives in response to the diverse infrastructure, logistics and services of the various healthcare facilities. Despite this, 48.2% of the participants expressed concerns, which mainly related to possible non-adherence of patients with chronic conditions, the lack of promotive and preventative services, challenges with facility infrastructure, and staff time devoted to triage and screening. More than half of the participants (57.8%) indicated that other services were affected by COVID-19, whilst 44.6% indicated that these services were worse than before.

**Conclusion:**

Our findings suggest that the very necessary reorganisation of services that took place at the start of the COVID-19 pandemic in South Africa enabled effective management of patients infected with COVID-19. However, the reorganisation of services may have longer-term consequences for primary care services in terms of lack of care for patients with other conditions, as well as preventive and promotive care.

## Introduction

Primary health care (PHC) is widely accepted as the cornerstone of universal health coverage.^1^ According to the 2018 Astana declaration, PHC is the most cost effective and inclusive means of delivering health services that can effectuate the well-being of all people.^2^ It is estimated that nurses, who represent 60% of the healthcare workforce,^3^ render about 90% of international primary care (PC) services.

Building into and reorganising PC services alongside developing effective strategies to reach underserved populations are key to strengthening the health system and have been shown to have positive effects on health outcomes.^4^ In South Africa, re-engineering of PHC towards universal health coverage^5^ was built on an existing framework of clinics and community health centres that provide first level care to the majority of the population. The reach of these facilities is significant, but the quality of care provided is vastly variable, and noted to be poor in many parts of the country.^6^

Primary care nurses, especially Clinical Nurse Practitioners, play a pivotal role in these frontline health facilities as they essentially work as substitutes for doctors.^7^ They are, inter alia, responsible for preventive and promotive healthcare (including child health and maternity care), diagnoses and treatment of common conditions, provision of ongoing management of patients with chronic illness, and the provision of support to community health workers, in line with trends in many other countries.^8^ Ideally, PC nurses should function within an effective PC team,^9^ but challenges in alignment between the PHC strategy and human resources for health lead to a very uneven realisation of that goal across the country.^10,11^ Furthermore, a disjuncture between the South African ‘Ideal Clinic’ and the Office of Health Standards Compliance’s criteria, two separate national quality care initiatives, puts strain on the resources of the provinces and the frontline staff because of diffused accountability and their lack of involvement in decision-making.^6^

Of relevance to the current context, PC nurses play a key role in the response to and management of infectious diseases, being the first point of entry into the healthcare system.^12^ This has resulted in PC nurses being increasingly expected to work long hours, with suboptimal nurse-patient ratios, and to upskill on very short notice to manage new types of diseases.^13^

The South African healthcare system is committed to the provision of various preventative and curative services, including chronic care. These important services had to continue, while PC staff managed additional workloads because of the COVID-19 pandemic.^14^ The Western Cape Department of Health advised that healthcare services should be reorganised to ensure that healthcare facilities could attend to people in need of urgent attention. For PHC services, the recommendations for reorganisation included: postponement of non-urgent outpatient appointments; stable chronic patients issued with two months’ supply of medication; reduced outreach support; and suspension of ‘Chronic Club’ activities.^15^ However, research on how the recommendations were applied in various PHC settings has not yet been published.

COVID-19 related reorganisation strategies include screening of the patients on arrival at a clinic. Patients are provided with a facemask and taught cough etiquette, whilst surface decontamination and hand hygiene are promoted. Suspected cases of COVID-19 should be rapidly triaged and placed in a separate waiting room, ideally in a well-ventilated space.^15,16^ However, PHC facilities are often overcrowded with limited space. Given the critical role of PC nurses in this context, there is also an urgent and clear need for strategies to protect, support and manage exposed and infected healthcare workers.^17,18^

In addition to the reorganisation of the clinical health services, COVID-19 also demanded strategies to reorganise management and leadership. Nurse leaders have commented on three missing aspects noticed in nursing leadership during the COVID-19 pandemic, namely the non-visibility of nurse leaders, a lack of collaboration amongst nurse leadership, and a failure to advocate for person-centred decision-making.^19^ The COVID-19 pandemic necessitated the development of innovative strategies on how to prevent and manage this new illness and continue with essential PC health services, especially in low resource settings where many people depend on public health services for treatment and care. In this testing and strenuous environment, a lack of leadership may negatively influence work performance, with ultimate poor mental health and increased anxiety about COVID-19-related issues.^20^ Reorganisation is therefore necessary to support staff as well as patients, and to ensure that the quality of services are maintained or improved, instead of being neglected.

It is unclear how the COVID-19 pandemic affected PC services in the Western Cape and what reorganisation strategies were employed. The aim of this study was thus to investigate the reorganisation of PC services in the Western Cape from the perspectives of PC nurses, to make context-appropriate recommendations for improving such processes during pandemics or other public health disasters.

## Methods

### Design

An exploratory-descriptive quantitative study was undertaken. The online survey was sent to Stellenbosch University’s Postgraduate Diploma in Primary Care Nursing students and alumni. This postgraduate diploma in Primary Care Nursing prepares nurses to assess, diagnose and manage a range of conditions in PHC settings. Admission criteria for a postgraduate diploma include at least two years of experience as a professional nurse. Students from various geographical locations in the Western Cape attend training at Stellenbosch University. The researchers had access to the contact details of these students and alumni, and they were therefore the accessible population for a rapid assessment at the time of the study which was conducted during the peak of the first wave of COVID-19 pandemic.

### Setting

The Western Cape province of South Africa is one of nine provinces and has a population of about 6.6 million people of which 64% reside in the City of Cape Town urban district. Three quarters (75.2%) of people in the province utilise PHC services.^21^ During 2019/2020, the Western Cape Department of Health reported 14.3 million PC encounters.^21^ The Western Cape has the highest life expectancy (68 years vs. 64 years on average for South Africa) and lowest maternal mortality rate (68.3 per 100 000 live births vs an average of 119 per 100 000 live births in South Africa) in the country.^21^

The three core services of the PHC platform in the Western Cape include: community-based care (via non-profit organisations and community health workers), PC (in 266 fixed and non-fixed facilities) and intermediate care. Primary care is driven by PC nurses and includes a range of services, including child and adult curative services, preventative services, women’s health, mental health, human immunodeficiency virus (HIV), tuberculosis (TB), and chronic disease management.^21^ The Postgraduate Diploma in Primary Care Nursing students and alumni that was the target population for this study also provide these services.

Primary care nurses include professional nurses with undergraduate diplomas and degrees working in PHC settings as well as those who have completed an additional Postgraduate Diploma in Primary Care Nursing (Clinical Nurse Practitioners) that enables them to assess, diagnose, prescribe treatment for, and manage persons according to the PC guidelines.^21^

### Instrument

We developed a questionnaire based on the Impact of COVID-19 on the Nursing and Midwifery Workforce study (ICON) questions^22^ and other relevant literature. The questionnaire was based on the structures and processes needed for preparedness for COVID-19 and included: demographic information, COVID-19 training and attitudes, access to guidelines, facilities and equipment, services reorganisation, information, and training needs and personal or self-care needs. The questionnaire was reviewed by four external experts, including two nursing health services managers, one academic involved in the COVID-19 response in the Western Cape, and one expert working for a non-governmental organisation involved in community education and testing in relation to COVID-19. The experts suggested some changes to a few questions, as well as some additional questions, but were overall happy with the content of the questionnaire. The final questionnaire comprised of 48 questions, both closed- and open-ended. Open-ended questions allowed for participants to provide explanations or comments regarding their context that may have not been captured by closed-ended questions. For this article, we focused on the questions related to services reorganisation.

Validity was ensured by developing the questionnaire from the literature and subjecting it to expert review. Reliability analysis could only be performed on the questions that measured similar concepts on a Likert-type scale. These questions related to confidence and preparedness (Cronbach alpha 0.7) and personal and self-care needs, specifically worry and anxiety (Cronbach alpha 0.75).

### Population and sample selection

Professional nurses enrolled for the Postgraduate Diploma in Primary Care Nursing (year 2020) and alumni from the years 2017–2019 were included (*n* = 251). We excluded nurses who were not working in PHC practice at the time of the study or who did not have working emails (*n* = 37). The total number of eligible participants was 214. We included the total population in the sample to account for non-responders. A minimum sample size 136 was needed for representativeness.^23^

### Pilot survey

A pilot survey was conducted to assess whether the questions were clear for participants and if they could easily follow the electronic link and complete the online questionnaire. We selected 20 students from the 2016 cohort of which 12 completed the questionnaire. We made a few adjustments to some questions and did not include the pilot data in the main study.

### Data collection

An email was sent to participants with a link to complete the questionnaire. Reminders were sent to participants who did not complete the questionnaire. Participants completed the questionnaires during the peak of the first wave of the pandemic, between 30 June 2020 and 01 September 2020. We sent a total of five reminders. Most participants completed the questionnaire in July, with few responses received thereafter. There was therefore only one wave of responses. We did not perform non-response analysis as we did not have access to the demographic details of the participants who did not respond.

### Data analysis

Data were analysed descriptively and summarised in frequency tables. Comparisons between participant responses and whether they were working in an urban or rural area were made using cross-tabulations and the Chi-square or Fisher’s exact statistics. Content analysis^24^ was used to analyse the open-ended questions and the frequencies of themes identified. The process involved at least two of the authors reading the responses, dividing the text into meaning units and formulating codes. The codes were then grouped into categories and overarching themes. Once all the responses were coded and linked to a theme, the frequencies of the themes for each response were calculated. Verbatim quotes were added to support each theme. All the authors reviewed the themes and quotes for meaningfulness and credibility. Quotes were labelled as follows: female or male (F/M), age and rural or urban district (R/U).

### Ethical considerations

We obtained ethical approval from the Health Research Ethics Committee at Stellenbosch University (N20/04/015_COVID-19) on 15 March 2020. Institutional approval from Stellenbosch University was provided to access the email addresses of students and alumni after signing an agreement outlining the *Protection of Personal Information Act* 4 of 2013 requirements. Participants could read the online information leaflet and voluntarily decide to participate. Responses were anonymous and not linked to participants’ information.

## Results

### Participants

Eighty-three participants completed questionnaires, a response rate of 38.8%. The mean age of participants was 37.8 years (range 27–55 years) and the mean number of years of working in PHC was 5.4 (range 0–20 years). Most participants (*n* = 49; 61.3%) worked in urban districts (Cape Metropole and City of Cape Town), while 31 (37.3%) worked in rural districts such as the Cape Winelands (*n* = 13; 41.9%), Overberg (*n* = 4; 12.9%), West Coast (*n* = 4; 12.9%), Eden (*n* = 6; 19.4%), and Karoo (*n* = 4; 12.9%). Three participants (3.6%) did not indicate a specific district. Participants worked in public health clinics (*n* = 32; 38.6%), community health centres (*n* = 26; 31.3), mobile clinics (*n* = 5; 6%), private clinics (*n* = 7; 8.4%) and other types of facilities (*n* = 16; 19.3%) such as correctional services, military service, home-based care, and non-governmental organisations.

**FIGURE 1:**
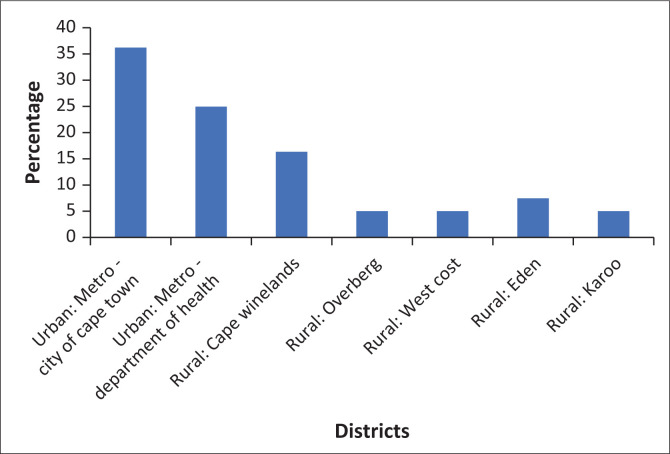
Districts participants were working in.

Figure 1 indicates the health districts in the Western Cape where the participants were working.

### Reorganisation strategies

A substantial majority of participants reported that they were reorganising services (*n* = 61; 74.4%), with half expressing concern about the reorganisation (*n* = 40; 48.2%). The most common service reorganisation strategy, based on the options provided in the survey, was issuing multiple months of supply of medication for those with chronic illnesses (*n* = 44, 53.0%), followed by postponement of non-urgent appointments (*n* = 37; 44.6%), and reduction of outreach support (30; 36.1%) (see Table 1). A minority of the participants (*n* = 24; 28.9%) indicated that they were continuing to provide non-COVID-19 acute care. Other initiatives being undertaken as reported by participants. Nine participants (10.8%) reported other initiatives. These initiatives included: (1) Chronic medication related such as electronic scripting before appointments, telephonic scripts, medication dispensing outside facilities, teaming up with an non-government organisations to do home deliveries of medications; (2) Patient appointments/flow improvements such as drawing patient files that allowed patients to leave earlier, telephonic consultations with healthcare providers prior to visiting the facilities, facilities divided to see sick children; and (3) Human resource related reorganisation such as obtaining help with immunisations and reproductive health and high-risk staff not working in isolation areas.

**TABLE 1:** Reorganisation of services.

Question/variable	Urban		Rural		Total	
	*n*	%	*n*	%	*n*	%
**Are you currently triaging or managing COVID-19 cases in the facility where you are working? (*n* = 83)**						
Yes	30	61.2	17	54.8	47	56.6
No	19	31.8	14	45.2	35	42.2
Missing	-	-	-	-	1	1.2
**How many PUI for COVID-19 have you had direct clinical contact with? (*n* = 83)**						
None	2	4.1	5	16.1	7	8.4
Less than 5	12	24.5	9	29.0	23	27.7
More than 5	14	28.6	2	6.5	16	19.3
Too many to count	21	42.9	15	48.4	36	43.4
Missing	-	-	-	-	1	1.2
**Do you have to reorganise or change the services in the clinic because of COVID-19? (*n* = 83)**						
Yes	38	77.6	21	67.7	61	73.5
No	11	22.4	10	32.3	21	25.3
Missing	-	-	-	-	1	1.2
**How are services being reorganised† (*n* = 83)**						
Stable chronic patients issued multiple months’ supply of medication	27	55.1	17	54.8	44	53.0
Non-urgent appointments are postponed, and patients are given alternative dates	19	38.8	16	51.6	37	44.6
Outreach support for example to schools or the community is reduced	16	32.7	13	41.9	30	36.1
We are continuing to provide acute care (excluding COVID-19)	12	24.5	11	35.5	24	28.9
Chronic club activities are suspended	10	20.4	5	16.1	15	18.1
We are redeploying healthcare workers	6	12.2	6	19.4	12	14.5
We are not providing well-baby services such as immunisations	2	4.1	0	-	2	2.4
We are not providing female reproductive health services such as family planning and pap smears	1	2.0	1	3.2	2	2.4
We are not providing psychiatric services	1	2.0	0	0.0	1	1.2
Any other	6	12.2	2	6.5	9	10.8
**Do you have any concerns about the way that the PHC services are currently organised to respond to COVID-19? (*n* = 83)**						
Yes	20	40.8	19	61.3	40	48.2
No	29	59.2	12	38.7	42	50.6
Missing	-	-	-	-	1	1.2

PHC, primary health care; PUIs, persons under investigation; COVID-19, coronavirus disease 2019.

† , Multiple response options so variables do not add up to 100%.

Some concerning strategies were also mentioned, which included minimising HIV testing services and stopping monthly weighing of babies.

There was a significant difference between the number of persons under investigations (PUIs) that participants in this study had direct contact with in rural versus urban areas, with participants in urban areas indicating higher numbers (Fishers Exact, *p* = 0.037) (see Table 1). No other variables related to services reorganisation showed significant differences between urban and rural areas.

Another form of reorganisation included infrastructure organisation such as equipment and supplies. Restructuring possibly led to a shortage of adequate equipment needed to triage and manage COVID-19 patients. Most of the participants (*n* = 42, 50.6%) disagreed or strongly disagreed that adequate infrastructure was available to triage and manage patients with COVID-19. A lack of personal protective equipment (PPE) was illustrated with only 49 (59.0%) participants agreeing or strongly agreeing that they had access to the correct PPE when treating patients with COVID-19 (Table 2).

**TABLE 2:** Infrastructure and equipment organisation.

Question/variable	Urban		Rural		Total	
	*n*	%	*n*	%	*n*	%
**There is adequate infrastructure in my facility to triage and manage COVID-19? (*n* = 83)**						
Strongly agree	5	10.2	2	6.5	7	8.4
Agree	10	20.4	8	25.8	18	21.7
Neither agree nor disagree	11	22.4	4	12.9	15	18.1
Disagree	16	32.7	9	29.0	27	32.5
Strongly disagree	7	14.3	8	25.8	15	18.1
Missing	-	-	-	-	1	1.2
**The correct PPE (as recommended by my employer) is always available to me when treating a patient with COVID-19 (*n* = 83)**						
Strongly agree	6	12.2	9	29.0	15	18.1
Agree	20	40.8	12	38.7	34	41.0
Neither agree nor disagree	12	24.5	2	6.5	14	16.9
Disagree	8	16.3	6	19.4	14	16.9
Strongly disagree	3	6.1	2	6.5	5	6.0
Missing	-	-	-	-	1	1.2

PPE, personal protective equipment; COVID-19, coronavirus disease 2019.

Open-ended responses revealed a multitude of restructuring initiatives that were undertaken in response to the diverse infrastructure, logistics and services of the various healthcare facilities. The restructuring initiatives were related to facility, operational and service restructuring. The facility restructuring included dividing the clinic into separate areas, creating a separate entrance for PUIs and creating isolation wards for positive COVID-19 patients as illustrated by the responses of following participant:

‘They have allocated separate entrance for COVID [*coronavirus disease*] suspects […].’ (Female, 33 years old, rural)

‘[…] we had to cut down our clinic into two sections to accommodate COVID-19 [*coronavirus disease 2019*].’ (Female, 40 years old, urban)

The lack of space in facilities resulted in the creation of extra space. This was done by establishing waiting areas outside or in the garage [*certain smaller clinics are situated in a domestic home structure*], use of gazebos and enlarging the reception areas:

‘Patients must wait in the garage in winter times or sit exposed to elements outside.’ (Female, 34 years old, rural)

‘We support with gazebos to facilities where there are space constraints for COVID-19 [*coronavirus disease 2019*] screening and testing.’ (Female, 31 years old, urban)

Other restructuring initiatives were related to COVID-19 screening and testing. Screening for COVID-19 was done outside the facility, in the pharmacy area or in containers on the facility’s premises. At some facilities, COVID-19 testing was done using the sputum booths and storerooms:

‘Night staff had to screen patients in a pharmacy area then to the cold container for the whole night.’ (Female, 39 years old, urban)

‘Our facility tests the suspect outside the facility … on clinic premises. The area is just barricaded with cardboard for some privacy and at times it’s done at the main entrance door, a shield is being used as a barrier for privacy.’ (Female, 35 years old, rural)

‘We currently do COVID [*coronavirus disease*] testing outside the facility in our sputum booth.’ (Female, 35 years old, rural)

Service restructuring related to operational functioning was linked to appointment changes which consisted of cancelling or rescheduling appointments. Patients were given long-term follow-up dates. Telephonic consultations were done to determine the necessity of an appointment. Patients with comorbidities were also informed to stay home unless they required urgent treatment:

‘Appointments had to be rescheduled, groups sessions had to be cancelled.’ (Male, 43 years old, urban)

‘Patients phone for their chronic repeat scripts … Date time given when to collect it. Minimum patients allowed at sickbay. Only emergency patients. Consult on phone and HCP [*health care professional*] will decide if it necessary to come in …’ (Female, 32 years old, no district – military services)

‘We are trying to give the message that those with co-morbidities to stay home and come to clinic when it is really necessary.’ (Female, 29 years old, rural)

The service restructuring was dependent on the facility. Table 3 indicates the services and procedures at primary and secondary levels of care that were stopped during the first wave of the pandemic.

**TABLE 3:** Services stopped during the COVID-19 pandemic.

Services stopped (*n* = 83)	Participant quotes	Frequency†	Percentage
Dental, physiotherapy, dietician, X-rays	‘Other essential services are cancelled like dental, physio, dietician, X-ray, this creates great difficulty in treating patient correctly.’ (M, 30, R)	10	11.7
Minor surgery/procedures	‘Minor OP theatre closed.’ (F, 51, U)	4	4.7
No weight checks	‘No routine weight checking of babies are done.’ (F, 35, R)	4	4.7
No family planning and infant immunisations	‘Most clinics do not offer well baby immunisations and family planning.’ (F, 29, no district – private clinic)	4	4.7
Eye clinic	‘Employees who have been referred have come back with notes stating the eye clinic is closed due to COVID-19.’ (F, 41, U)	3	3.5
Outpatients department services suspended	‘OPD appointments deferred.’ (F, 50, U)	3	3.5
Occupational therapy	‘Services suspended including OT.’ (F, 34, R)	2	2.3
No aerosol procedures	‘Aerosol procedures are being avoided e.g. Spirometry tests.’ (F, 39, U)	2	2.3
Pap smears not done	‘Pap smears are not done and this is particularly concerning as early detection of cancer will be missed.’ (F, 36, R)	2	2.3
HIV services	‘HIV testing services have been minimised, including index contact tracing.’ (F, 31, U)	1	1.1
Social services	‘As HBC nurse, it is frustrating because we end up with more cases. Social services just say they are on lockdown.’ (F, 46, U)	1	1.1
Not all bloods were routinely done	‘No bloods are done routinely on the chronic patients. Only INR patients’ bloods are drawn.’ (F, 35, R)	1	1.1

OP, operating theatre; OPD, out patient department; OT, occupational therapy; HBC, home-based care; HIV, human immunodeficiency virus; COVID-19, coronavirus disease 2019; INR, international normalised ratio; F, female; M, male; R, rural; U, urban.

† , Frequencies and percentages were calculated out of the number of participants who responded and represents the frequency of the themes in the participant narratives.

### Consequences of and concerns related to service restructuring

In the open-ended questions, participants were asked about their concerns related to the reorganisation of services. Table 4 depicts the most common themes related to these concerns. The greatest concerns were related to possible non-adherence of patients with chronic conditions, challenges with facility infrastructure and staff time devoted to triage and screening.

**TABLE 4:** Concerns related to services reorganisation.

Themes (*n* = 83)	Example quote	Frequency†	Percentage
Chronic condition defaults	‘Afraid that there will a high rate of ARV defaulters, MDRs and high rate of patients with sensitive TB after all this.’ (F, 47, U)	11	13.2
Infrastructure problems	‘Infrastructure and outlay of building not suitable.’ (F, 40, U)	8	9.6
Screening and triaging difficulties	‘Personnel needs to be there to triage, while the rest needs to see the other patients. Which means if the triaging and testing of COVID testing are done there sometimes is a long waiting time for the rest of the patients.’ (F, 29, R)	6	7.2
Staff burnout	‘Burn out for staff as we are divided now. High risk of staff going off sick.’ (F, 34, U)	3	3.6
Continuation with regular services	‘Stable chronic patients that are still coming to clinic, club patients still attending as usual with their active services not cancelled.’ (F, 36, U)	3	3.6
Lack of leadership	‘Lack of leadership.’ (M, 47, U)	2	2.4
Staff shortages	‘Too little staff. I need to do COVID screening and testing and see to patients coming for normal acute and chronic conditions.’ (F, 39, U)	2	2.4
Non-holistic care provision	‘Care feels rushed and not holistic, because all focus is on COVID-19.’ (F, 34, R)	1	1.2
Insufficient COVID-19 precautions	‘Despite not having any positive COVID patients yet in the district I personally feel that stronger precautions should be implemented.’ (F, 30, R)	1	1.2
Lack of staff screening	‘We only completed the vulnerable forms and bring medical report to show that I have chronic condition, but no scoring done.’ (F, 46, U)	1	1.2

TB, tuberculosis; ARV, antiretroviral drug(s); MDR, multi drug resistant (tuberculosis); F, female; M, male; U, urban; R, rural.

† , Frequencies and percentages were calculated out of the number of participants who responded and represents the frequency of the themes in the participant narratives.

Service restructuring affected the staff, patients and the quality of care rendered. It also highlighted infrastructure, leadership, and management inadequacies.

Participants had concerns about potential communicable disease outbreaks as a result of immunisation services that were temporarily stopped. There were also concerns that patients’ conditions would worsen or they would default on their treatment regimens because of the changes made to service delivery:

‘When will all the children be immunised? What if another outbreak of disease appears?’ (Female, 53 years old, rural)

‘Minimum and limited services are being provided, less amounts of times spent on TB/HIV [*tuberculoses/human immunodeficiency virus*] patients mean they might develop complications that are not readily picked up in time, they might easily fall off the radar and become loss to follow.’ (Male, 37 years old, rural)

In response to service restructuring, patients experienced emotional distress. They were also afraid that they will not be helped, and this resulted in them being dishonest during the screening process:

‘We have a dedicated COVID [*coronavirus disease*] screening site and a COVID ward for stable COVID positive patients. However, this has greatly impacted the stable and unstable chronic patients and they are being given long term follow up dates. Thus, has caused some patients emotional stress.’ (Female, 51 years old, urban)

‘Screening not reliable as clients lie about being contacts because of the stigma and they are afraid that they will not be helped if they tell the truth that they were contacts of positive cases.’ (Female, 29 years old, urban)

The changes made during the pandemic affected the staff in many ways. Staff experienced burnout as a result of heavy workloads and a shortage of staff. Some facilities had to close temporarily because of all of the staff testing positive for COVID-19. Some participants felt that their working conditions were not safe and experienced increased levels of stress and anxiety:

‘There is a shortage of staff. Staff are overwhelmed and burnout is evident.’ (Male, 43 years old, urban)

‘Stress from daily worries of the possibility that we are at high risk of becoming the next statistic.’ (Female, 35 years old, rural)

‘Our onsite BANC [*basic antenatal care*] closed for a week as all staff had COVID [*coronavirus disease*] positive results.’ (Female, 51 years old, urban)

The restructuring in some instances influenced the quality of care rendered. While some patients experienced longer waiting times, others were fast tracked to minimise the risk to staff. At times, the lack of equipment and PPE compromised the care provided:

‘Triaging of patients is not done well, because patients will pass the gate to come into the clinic with no possible signs of COVID [*coronavirus disease*] only to find out when you consult them that they have either a cough or sore throat. Patients unfortunately crowd outside the clinic in a long queue.’ (Female, 35 years old, rural)

‘Mismanaging of other chronic patients as we fast track to minimise risk at the facility.’ (Female, 44 years old, rural)

‘In case of emergency and a patent have code blue the identified roos [*routes*] doesn’t have all necessary equipment available, for example, defibrillator, ventilators, etc.’ (Female, 33 years old, rural)

Facility infrastructure made it difficult to assign designated areas for COVID-19 positive cases and for PUIs. This resulted in ineffective isolation and infection and prevention control practices:

‘There’s no emergency room for suspected persons. There’s no toilet for suspected persons. There’s no waiting area for suspected persons. There is no specific area for treating patients with COVID-19 [*coronavirus disease* 2019].’ (Female, 28 years old, rural)

‘Infrastructure and outlay of building not suitable to facilitate no cross infection and contamination of facility and patients.’ (Female, 40 years old, urban)

Leadership and management inadequacies were highlighted as a result of the restructuring. Some participants felt that there was a lack of leadership and poor decision-making:

‘There is no decisive action taken regarding how to handle COVID [*coronavirus disease*] PUI’s [*persons under investigation*] or cases.’

### Redeployment

Healthcare workers who may be at high-risk should be identified and provided with the option for redeployment. In our sample, no risk score was calculated for 19 participants (23.2%). Of those who had a high-risk score, 22 participants (26.8%) were not provided with the option of redeployment (see Table 5).

**TABLE 5:** Redeployment.

Question/variable	Urban		Rural		Total	
	*n*	%	*n*	%	*n*	%
**Was your COVID-19 risk score calculated by yourself or your manager or Occupational Health practitioner? (*n* = 83)**						
Not calculated	14	28.6	4	12.9	19	22.9
By myself	9	18.4	14	45.2	24	28.9
By my manager	22	44.9	12	38.7	34	41.0
By an Occupational Health practitioner	3	6.1	1	3.2	4	4.8
Missing	-	-	-	-	2	2.4
**If your COVID-19 risk score was calculated as being high or unacceptable, were you given the option by your line manager to be redeployed? (*n* = 83)**						
Yes	9	18.4	4	12.9	13	15.7
No	12	24.5	9	29.0	21	25.3
Not applicable	27	55.1	18	58.1	47	56.6
Missing	-	-	-	-	2	2.4

COVID-19, coronavirus disease 2019.

### Influence on the other healthcare services and quality of healthcare

The majority of the participants (*n* = 48; 57.8%) indicated that other services were affected by COVID-19, whilst more than half (*n* 51; 61.4%) reported that there were fewer patients at the facility. Almost half of the participants (*n* = 37; 44.6%) indicated that the services were worse than before for other patients (Table 6).

**TABLE 6:** Services quality.

Question/variable	Urban		Rural		Total	
	*n*	%	*n*	%	*n*	%
**Is the COVID-19 pandemic currently affecting services rendered to patients with other conditions? (*n* = 83)**						
Yes	31	63.3	16	51.6	48	57.8
No	18	36.7	15	48.4	34	41.0
Missing	-	-	-	-	1	1.2
**How is the quality of care provided to patients who are NOT presenting with either symptoms or a diagnosis of COVID-19? (*n* = 83)**						
Significantly worse than before COVID-19	10	20.4	4	12.9	15	18.1
Slightly worse than before COVID-19	11	22.4	10	32.3	22	26.5
The same as before COVID-19	20	40.8	10	32.3	30	36.1
Slightly better than before COVID-19	3	6.1	4	12.9	7	8.4
Significantly better than before COVID-19	5	10.2	3	9.7	8	9.6
Missing	-	-	-	-	1	1.2
**Have your professional working conditions changed since the COVID-19 outbreak? (*n* = 83)** **Select all that apply** †						
More patients at facility	14	28.6	12	38.7	27	32.5
Fewer patients at facility	31	63.3	19	61.3	15	61.4
Shorter work hours	7	14.3	3	9.7	11	13.3
Longer work hours	3	6.1	4	12.9	7	8.4
Fewer breaks	7	14.3	10	32.3	17	20.5
More breaks	2	4.1	2	6.5	4	4.8
**Have you had to self-isolate or quarantine yourself at home? (*n* = 83)**						
Yes	35	71.4	14	45.2	50	60.2
No	14	28.6	17	54.8	32	38.6
Missing	-	-	-	-	1	1.2

COVID-19, coronavirus disease 2019.

† , Multiple response options so variables do not add up to 100%.

Suggestions provided by the participants on how to improve the circumstances created by the pandemic and the restructuring are depicted in Table 7.

**TABLE 7:** Suggestions for improvement.

Improvements (*n* = 83)	Quote	Frequency†	Percentage
Improve psychosocial support	‘I feel that government should value nurses and doctors all healthcare professionals by wellness programmes. More support and guidance for the mental aspect of COVID-19. Not forgetting recognition of staff.’ (F, 40, U)	19	22.3
More staff assistance	‘More staff should be made available because of staff shortages.’ (F, 33, R)	19	22.3
More PPE	‘My employer must provide proper PPE all the time because other patients with COVID-19 don’t show symptoms early.’ (F, 40, U)	18	21.1
Danger allowance	‘[…] pay the health workers risk allowance.’ (F, 36, U)	8	9.4
More training	‘When to wear what PPE must be revised as doctors and nurses don’t know whether or not patients have COVID-19 and they resus [*resuscitating patients*] on patients only to find out later that the patient is COVID-positive.’ (F, 33, R)	6	7.0
Improved infrastructure	‘Infrastructure that is conducive e.g. having taps in the office.’ (F, 48, R)	3	3.5
More equipment	‘More electronic thermometers.’ (F, 51, R)	2	2.3
Staff testing at facilities	‘[…] to allow us to test in our facilities for COVID-19 because currently we have to test privately and pay for COVID test which is expensive; my fear is just that when I get really sick and my funds will be depleted.’ (F, 34, U)	2	2.3
Leadership and effective health and safety practices	‘Strong nurse leaders. Attending to health and safety on mobile clinics.’ (F, 53, R)	1	1.1
Adherence to national policies	‘By adhering to national guidelines as stipulated, only seeing emergencies and booked appointments.’ (F, 30, R)	1	1.1

PPE, personal protective equipment; F, female; M, male; R, rural; U, urban.

† , Frequencies and percentages were calculated out of the number of participants who responded and represents the frequency of the themes in the participant narratives.

## Discussion

In alignment with what is happening internationally, healthcare workers and PC nurses demonstrated resilience to adapt and manage COVID-19 whilst continuing essential services.^25,26^ The required reorganisation of services mandated provincially was differentially applied at a local level, and this came at a cost to both patients and PC nurses. A study from Australia^27^ reported that PC reorganisation efforts for COVID-19 resulted in fewer face-to-face consultations but an increase in additional tasks incorporated in the nursing role. Furthermore, a similarity was seen with the increase in work hours as reported by 8.4% of the participants and fewer breaks by 20.5% in this study.

Although almost half of the sample (43.9%) had seen ‘too many COVID-19 patients to count’ the majority reported seeing fewer patients at the facility, which could be related to the necessary reorganisation of services. While reorganisation allows for effective triaging, and keeping vulnerable patients out of harm’s way, the effects on patients, and other people needing care, is not known. Despite participants’ concern for the way that services were reorganised, individual creativity to provide care to those in need of it was described. Nyasulu and Pandya^14^ raised concerns about the impact of the pandemic on HIV care, the Expanded Programme on Immunization (EPI) and other essential services in the South African healthcare system. Blanchett et al.^18^ mentioned that a decline in essential services across lower- and middle-income countries may reverse health gains. Nyasulu and Pandya^14^ offered the building blocks of the World Health Organization’s (WHO) health systems framework as an approach for analysing and prioritising services, while Blanchett et al.^18^ provided a model list of 120 essential non-COVID-19 health interventions, based on the Disease Control Priorities-3 highest priority package. Whichever approach is used, it is clear that the PC nurses at the frontline are concerned about gaps in care and want to be involved in addressing these gaps.

Two major issues mentioned by PC nurses were poor infrastructure in which to provide care and the difficulty to perform screening and triaging because of staff shortages. While the intention was for reorganisation to allow two streams of patients (PUIs versus those thought not to be affected by COVID-19) in order to still provide essential services,^28^ in practice this was often suboptimal. Good communication and visible frontline support by managers and supervisors are needed.^29^ Butts and Rich^30^ mentioned that it is the responsibility of ethical leaders and governments to maximise preparedness in order to minimise the need to make allocation decisions later during a pandemic. Investment in robust public health infrastructures and health equity are the best preparation for dealing with a health disaster, such as a pandemic.^30^ Leaders therefore need to be organised, creative and adequate in their leading, and influence their employees as followers, as well as the public, to be involved with improving people’s health.

In response to addressing their own needs for safety and self-care, with a proportion of the participants having to perform risk-scoring themselves, some not checked at all, and for a number of participants not being given the choice to be redeployed was a concern. Clear strategies are needed to support and manage exposed and infected healthcare workers to ensure effective staff management and to foster trusting relationships in the workplace.^17^ Chersich et al.^29^ mentioned the importance of prioritising support for healthcare workers, suggesting 10 key interventions based on the literature that can make a difference in securing the health and mental well-being of frontline healthcare workers in the COVID-19 response in Africa. It is therefore important that the health and mental well-being of frontline workers form an integral part of reorganisation strategies amidst a pandemic.

## Strengths and limitations

Targeting only the Postgraduate Diploma in Primary Care Nursing students and alumni, the low response rate and possible response bias limits the generalisability of the findings. The demographic profile of the participants reflects the vast age profile of healthcare workers in the province (85% of healthcare workers in the Western Cape province are between the ages of 25 years and 55 years).^21^ Further, there were no differences across subgroups and the results were in accord with our own experiences and anecdotal reports, which suggest validity and credibility. In addition, qualitative data provided by participants and the similarities between our study findings and what was found in other settings enhances the credibility of the results.

## Conclusion

Primary care services are pivotal in the pandemic response. Our findings suggest that the very necessary reorganisation of services that took place at the start of the COVID-19 pandemic in South Africa enabled effective management of persons infected with COVID-19. However, the reorganisation of services may have longer-term consequences for PC services in terms of lack of care for patients with other conditions, as well as preventive and promotive care, that will only be seen in time. It is encouraging that the PC nurses are aware of this issue and will thus hopefully act to address it going forward, but it is possible that the damage has been done and cannot be reversed. Similarly, the resilience and goodwill that seem to exist, need to be strengthened and harnessed going forward, which requires implementation of some of the interventions we have described, both in terms of human resource management and system restructuring. The study highlights leadership, management, staff support, infrastructural and equipment deficits in PHC settings that should be addressed to realise the vision of universal health coverage.
